# Dazzle camouflage, target tracking, and the confusion effect

**DOI:** 10.1093/beheco/arw081

**Published:** 2016-05-31

**Authors:** Benedict G. Hogan, Innes C. Cuthill, Nicholas E. Scott-Samuel

**Affiliations:** ^a^ Biological Sciences, University of Bristol, Bristol Life Sciences Building, 24 Tyndall Avenue, Bristol BS8 1TQ, UK and; ^b^ Experimental Psychology, University of Bristol, 12a Priory Road, Bristol BS8 1TU, UK

**Keywords:** confusion effect, dazzle camouflage, defensive coloration, target tracking.

## Abstract

The influence of coloration on the ecology and evolution of moving animals in groups is poorly understood. Animals in groups benefit from the “confusion effect,” where predator attack success is reduced with increasing group size or density. This is thought to be due to a sensory bottleneck: an increase in the difficulty of tracking one object among many. Motion dazzle camouflage has been hypothesized to disrupt accurate perception of the trajectory or speed of an object or animal. The current study investigates the suggestion that dazzle camouflage may enhance the confusion effect. Utilizing a computer game style experiment with human predators, we found that when moving in groups, targets with stripes parallel to the targets’ direction of motion interact with the confusion effect to a greater degree, and are harder to track, than those with more conventional background matching patterns. The findings represent empirical evidence that some high-contrast patterns may benefit animals in groups. The results also highlight the possibility that orientation and turning may be more relevant in the mechanisms of dazzle camouflage than previously recognized.

## Lay Summary

The influence of coloration on the ecology of moving animals in groups is not well understood. High-contrast stripes may act as “motion dazzle camouflage” by preventing predators from accurately tracking prey, particularly in groups. We used a computer study with human “predators” to investigate this and found that targets in groups with horizontal striped patterns were harder to track than the ones with conventional camouflage, and the benefit increased with group size.

Twitter: @Camo_Lab

## INTRODUCTION

Two common solutions to avoiding predation in animals are coloration and aggregation (Ruxton et al. 2004). However, only in the context of warning coloration—largely because of the implication of family grouping effects—has there been much investigation of the interaction between the two (Gamberale and Tullberg 1996, 1998; Hatle and Salazar 2001; Riipi et al. 2001). This article addresses the potential synergy between so-called “dazzle camouflage” and the confusion effect, each an influential idea in its own right but rarely considered together (Hughes et al. 2015).

Although the first lines of defense of many animals are avoidance of detection and recognition, surface coloration has been proposed to have a role, once the prey is detected, in minimizing the probability of successful attack. Thayer (1909) hypothesized that highly salient, contrasting patterns may disrupt an observer’s perception of the trajectory or speed of a moving animal. This idea, perhaps convergently, was also advanced by military camouflage designers during both world wars, becoming known as “dazzle camouflage” (Behrens 1999, 2002). The ability to disrupt an observer’s perception of movement could confer a great selective advantage because accurate tracking may be vital to prey capture, or conversely to prey escape (Stevens et al. 2008, 2011; Scott-Samuel et al. 2011; Hughes et al. 2014; Hämäläinen et al. 2015; Hughes et al. 2015).

There is some evidence to support this hypothesis. How and Zanker (2014) used a biologically motivated motion detection algorithm to investigate signals elicited by the movement of images of the common plains zebra (*Equus burchelli*). Their findings supported the hypothesis that striped patterns generate aberrant movement signals in insect and mammalian vision systems. In addition, carefully controlled behavioral research on human participants has indicated that dazzle camouflage may disrupt speed perception. Scott-Samuel et al. (2011) found that some dazzle patterned objects were perceived as moving more slowly than plain objects. Furthermore, a computer-based game-like experiment by Stevens et al. (2011) found that high-contrast pattern targets were “caught” by human participants significantly less often than camouflaged targets.

However, the ways in which camouflage affects the capture of moving individuals in groups is poorly understood (Hall et al. 2013). Such individuals may experience reduced predation through a number of mechanisms, including dilution and vigilance effects (Krause and Ruxton 2002). Another mechanism is the “confusion effect,” which describes reduced predator attack success with increasing prey group size (or density; Ioannou et al. 2009; Scott-Samuel et al. 2015). There is good evidence for this effect in a variety of biological settings and in varied taxa (for a review, see Jeschke and Tollrian 2007). This effect may be driven by the increased cognitive challenge of selecting or tracking one moving individual among many (Krakauer 1995; Ruxton et al. 2007). Increases in group size may increase the attentional resources required for target tracking; this could be compounded by the effects of dazzle camouflage because these targets are individually more difficult to track. This would represent an advantage to animals that are found in groups and have plausible dazzle coloration, for instance those fish where stripes and shoaling have been found to be associated (cichlids, family: Cichlidae, Seehausen et al. 1999; but see butterflyfish, family: Chaetodontidae, Kelley et al. 2013).

A recent study by Scott-Samuel et al. (2015) utilized a computer-based task in which human participants tracked 1 target object among many distractor objects to investigate the effects of group size, density, and predictability of movement on the confusion effect. In the present study, this paradigm was adapted to test systematically the influence of dazzle and background matching camouflage and the confusion effect on object tracking.

Stripe patterns orthogonal or parallel to the direction of motion were applied to the target and distractor squares in order to investigate the influence of stripe orientation on motion dazzle effects. A recent study by Hughes et al. (2015) found that single orthogonally or obliquely striped patterned targets and plain targets were harder to catch than parallel striped targets. The authors also tested participants’ ability to capture groups of targets and found that striped targets in groups were actually easier to catch than plain targets, despite opposite predictions. However, this study and several previous studies on dazzle camouflage (Scott-Samuel et al. 2011; von Helversen et al. 2013; Hughes et al. 2014) have utilized targets that move in straight lines. In contrast, the current study uses targets and distractors that move unpredictably. Unpredictable (or protean) movement is a relevant feature to include in this investigation because it is an escape strategy has been found in a variety of taxa (Humphries and Driver 1970), and is known to enhance the confusion effect (Scott-Samuel et al. 2015; although see Jones et al. 2011).

Another key difference between this and previous research lies in the nature of the task. Most previous research has asked participants to click with a mouse or touch a touch screen to “capture” targets. Although this paradigm is perfectly consistent with the definition of the confusion effect (“the reduced attack-to-kill ratio experienced by a predator resulting from an inability to single out and attack individual prey”; Krause and Ruxton 2002), it does not isolate the effect on target tracking. This is because participants in such studies are free to use any strategy to capture prey, which may or may not require continuous or accurate tracking. The current study required participants to track a moving target for the whole duration of the trial and used tracking error as the dependent variable. This increases the chance of uncovering any effects that motion dazzle may have on target tracking per se. If high-contrast geometric patterns inhibit tracking in line with the suggestions of dazzle camouflage, we predict that the tracking errors for orthogonally and parallel striped targets will be larger than for the background matching ones. In addition, if dazzle camouflage can enhance the confusion effect, we would expect steeper relationship between group size and tracking errors for striped targets than background matching ones. Finally, differences in tracking between the results for orthogonally and parallel striped targets may help us to understand the mechanism or mechanisms behind dazzle camouflage.

## METHODS

A computer-driven task was created in Matlab (The Mathworks Inc, Natick, MA) using the Psychophysics Toolbox extensions (Brainard 1997; Pelli 1997; Kleiner et al. 2007). All stimuli were viewed at 62cm from a gamma-corrected 19″ Dell Trinitron CRT monitor, with a refresh rate of 100 Hz, a resolution of 1024×768 pixels, and mean luminance of 71.4 cd/m^2^. At the experimental viewing distance, each pixel subtended 2.2 minarc.

On each trial, subjects were presented with sets of 1, 10, 20, 30, 40, 50, or 60 moving squares, which were constrained within a central area on the screen (268×268 pixels). Each square was 32×32 pixels in size and moved at 200 pixels/s (7.54 visual degrees/s). The direction of movement of all squares from one frame to the next can be described as a correlated random walk. The direction of movement of each square in each frame was random, but with weighted probabilities that were described by a circular Gaussian distribution, such that continuing in the same direction was the most probable and more extreme deviations were less probable. The standard deviation of the circular Gaussian distribution was fixed at π/8 radians in this experiment, a value that was selected from pilot studies. In the current study, the orientation of the squares matched their direction of movement, such that each square maintained a constant orientation relative to its heading, which allowed the investigation of the effect of oriented color patterns.

**Figure 1:**
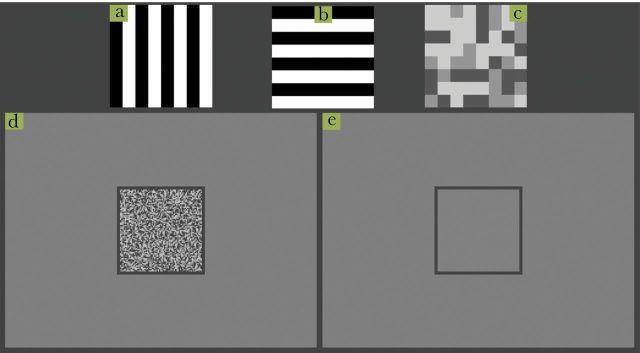
Illustration of the stimuli set used; (a) square pattern with bands orthogonal to the horizontal direction of movement, (b) square pattern with bands parallel to the horizontal direction of movement, (c) trinary square pattern, (d) example of screen with trinary background, and (e) example of screen with mean luminance background.

In each trial, the background on which the objects were drawn was either the mean luminance of the targets (71.4 cd/m^2^, see Figure 1e) or a trinary noise pattern, of the same mean, with each element (4×4 pixels) having a luminance of 35.7, 71.4, or 107.1 cd/m^2^ (with probability 1/3, see Figure 1d). The reason to include the trinary noise pattern was to allow for a treatment in which the targets matched the background perfectly, but were still detectable when moving. Had the targets and background been plain gray, they would be invisible (actually nonexistent) even when moving, an unrealistic situation given that most backgrounds are textured and motion breaks simple background matching camouflage (Hall et al. 2013).

There were 3 coloration treatments applied to the moving squares: one was a trinary pattern that matched the background (see Figure 1c), and the other two were made up of a 100% contrast square-wave grating with wavelength 8 pixels, oriented either parallel or orthogonal in relation to the square’s motion (see Figure 1a,b). The phase of the square-wave (starting black-white or vice versa) was randomly assigned as 0° or 180° with probability 1/2. Each combination of background and square coloration was combined factorially to form 6 conditions.

The participant’s task was to track the movements of the target square with a mouse-controlled on-screen cursor (a red circle, so as to provide clear discrimination from the targets and background, with an 8 pixel radius) until the end of a 5000-ms moving period. One of the squares was highlighted for 1000ms at the onset of each trial, indicating that this was the target square. The Cartesian locations of the center of the target square and center of the cursor were recorded every 10ms. The mean distance of the cursor from the target in pixels for the final 4000ms of each trial was calculated and recorded. Each participant completed 4 practice trials that were excluded from the analysis, followed by 336 trials in 6 randomly ordered blocks, one for each combination of coloration condition and background condition. The order of trials within each block (the order of presentation of the group sizes) was also randomized independently for each subject. There were 16 participants, who were recruited opportunistically, and each was reimbursed £7 for participation. Each gave their informed written consent in accordance with the Declaration of Helsinki, and the experiment was approved by the Ethics of Research Committee of the Faculty of Science, University of Bristol.

All statistical analyses were performed in R (R Foundation for Statistical Computing, www.R-project.org). Participant mean response errors were distributed approximately log-normally, so were transformed with a natural logarithm for all analyses, which utilized general linear mixed models (function lmer in the lme4 package; Bates et al. 2015). Relaxing the compound symmetry assumption for this repeated measures design, by use of generalized least squares (function gls in package nlme; Pinheiro et al. 2015), produced a very similar result in terms of effect sizes and statistical significance, so we present the simpler analyses here. The most complex model fitted number of distractors as a quadratic polynomial, along with the 2 factors, target coloration type and background type. The first model includes the 3-way interaction of these factors, and subsequent models address whether main or interaction effects can instead be modeled as linear terms. A 3-way interaction between number, background, and coloration type would indicate that the efficacy of coloration types in influencing the confusion effect is dependent on background. Lacking a 3-way interaction, a 2-way interaction between any factor and number would indicate that the factor influences the confusion effect. These models were compared using the Akaike information criterion (AIC); however, for the benefit of those readers who are less familiar with model selection, a traditional Anova analysis can be found in the Supplemental Material.

## RESULTS

A model that fitted number as a quadratic polynomial was a better fit than one that fitted number linearly (AIC −565.236 vs. −345.185) so all subsequent models used a quadratic fit of number. The 2 best models on information theoretic grounds were models 3 and 4 (see Table 1). Model 3 contained all main effects, as well as the target × number interaction, and the background × number interaction. Model 4 contained all main effects, but only the target × number interaction term. Because the models are not distinguishably different, the latter model is preferred as it contains fewer terms. Thus, model 4 can be considered the most parsimonious description of the data.

**Table 1:** Table of fitted models^a^

Model	Fixed effects	df	AIC
0	Target, background, number, target × background × number, target × background, target × number, background × number	14	−345.185
1	Target, background, number, target × background × number, target × background, target × number, background × number	20	−565.236
2	Target, background, number, target × background, target × number, background × number	16	−563.116
3	Target, background, number, target × number, background × number	14	−576.61
4	Target, background, number, target × number	12	−576.328
5	Target, background, number	8	−573.58
6	Target, background, number, target × number	9	−584.852

^a^Model 0 contained number as a linear fit, all subsequent models fitted number as a quadratic. In model 6, the factor target was recoded to treat orthogonally striped and trinary targets identical. Participant was a random effect in all fitted models. df, degrees of freedom.

**Figure 2:**
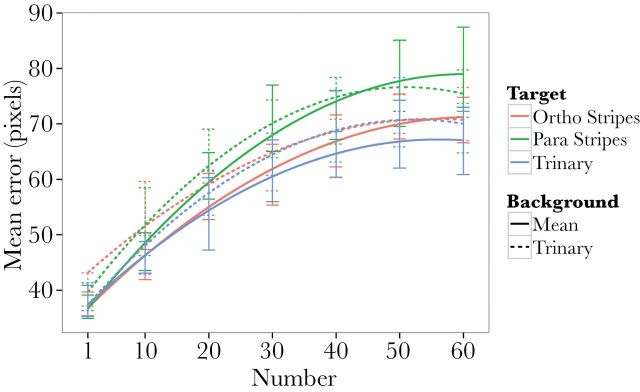
Quadratic fit of participant error against number of squares (target + *n* distractors), with color indicating target coloration condition and line solidity indicating background condition. Error bars indicate within subject 95% confidence intervals.

Because of a clear trend for increased targeting error in parallel striped conditions (see Figure 2), we next tested whether a model that assumes that orthogonally striped and trinary targets show identical effects would describe the data as well as the previous model. A model that assumes orthogonal stripes and trinary patterns are identical was a better fit that one that does not (AIC −584.852 vs. −576.328). Thus, the most parsimonious model was one where there is a difference in the quadratic effect of number between parallel striped targets and the combination of trinary and orthogonal striped targets (because this model has statistically indistinguishable explanatory power, but fewer terms).

## DISCUSSION

In this study, we present evidence that high-contrast dazzle coloration is superior to background matching camouflage in disrupting visual tracking of one target within a group. The results suggest that high-contrast stripes parallel to the direction of motion caused a significant enhancement of the confusion effect (an increase in tracking errors with distractor number) relative to the orthogonally striped or trinary noise patterns. This finding indicates that in principle, there may be situations in which a high-contrast striped pattern will benefit group-living animals.

The slopes of error against distractor number for the parallel striped conditions were steeper than for trinary or orthogonally striped targets. This indicates that there was a greater “confusion effect” (deleterious effect of group size on target tracking) for parallel striped targets. This supports the hypothesis that the increased attentional challenge that may cause the confusion effect can be enhanced by dazzle coloration, reducing an observer’s ability to track a given individual—but this does not generalize to any high-contrast pattern. Animals that move in groups may accrue greater benefits from parallel striped dazzle coloration than solitary prey, which could have implications for the understanding of the interface between coloration, movement, and group behavior. The analysis of correlations between ecology and pattern is relevant here. For example, a phylogenetically controlled analysis of the evolution of various patterns on cichlids in African lakes by Seehausen et al. (1999) found that the evolution of horizontal bar patterns was associated with piscivorous feeding modes and shoaling behavior. This is consistent with such a pattern having an advantage for animals which could benefit from confusing observers as to their movement, and which occur in groups. However, in a phylogenetically controlled analysis of coloration in butterflyfishes (Family: *Chaetodontidae*), Kelley et al. (2013) found no effect of sociability on the evolution of vertical or horizontal stripes. Nevertheless, the current findings do strongly suggest that it is unwise to assume that color patterns that correlate with sociality or group size must serve social functions. We have shown that some patterns may have specific nonsocial effects in groups of animals, and the current findings may similarly predict correlations between some striped color patterns and social group size.

The slopes of tracking error against number for the orthogonally striped condition were shallower than those of the parallel stripe on mean luminance background condition, indicating a reduced interaction with the confusion effect. Indeed, we found that a model that assumed that trinary and orthogonally striped patterns were identical was more parsimonious than one that did not. This is an important result because the only difference between the 2 high-contrast targets was the orientation of the pattern relative to the object’s movement. The orientation of the pattern relative to the movement of targets has only recently been addressed in behavioral investigations of dazzle camouflage and the confusion effect (von Helversen et al. 2013; Hughes et al. 2015). The theoretical predictions for the optimal orientation depend on the hypothesized mechanism or mechanisms that underlie dazzle camouflage’s efficacy. Spatiotemporal aliasing or “wagon wheel” arguments may predict that maximal motion dazzle would be caused by stripes orthogonal to movement, whereas “barber pole” or aperture problem arguments may suggest that stripes diagonal to movement may be most effective, by creating motion signals that diverge from the real vector of motion (How and Zanker 2014; Kelley LA and Kelley JL 2014). In the current experiment, tracking was impeded to a greater degree by targets with stripes that were parallel to the target’s movement than by those with stripes that were orthogonal to movement. Our results may highlight an underestimated importance of rotation and orientation in the mechanisms of dazzle camouflage. In contrast to many previous studies on dazzle camouflage, the motion of targets and distractors in this experiment was unpredictable, with turns and arcs, which may mean that accurate perception of the orientation and turning angle of the target was key in tracking. The proposed low-level mechanisms for motion dazzle would predict that parallel stripes will only create spurious motion signals when the target rotates. In contrast, these mechanisms predict that orthogonal stripes could produce spurious motion signals not only when rotating but also when moving linearly. It may be that it is the variability in the noise produced by parallel striped patterns that interrupted tracking. The timing of these aberrant motion signals may also be of interest because they will occur in synchrony with changes in direction which may also increase the likelihood of losing track of the target and, in group scenarios, the misidentification of individuals.

This study represents the first clear evidence of differential relationships between the confusion effect and target coloration, and suggests that some high-contrast patterns may benefit animals in groups to a greater degree than background matching ones. Interestingly, it was linear striped patterns oriented parallel to the direction of movement of the targets that caused the greatest tracking errors. This finding indicates that the orientation of the pattern in relation to movement is important and, in contrast to some recent findings, such stripes could aid predator defense. The inclusion of protean movement may have been key here because, in contrast with other motion dazzle studies, accurate perception of orientation and rotation rather than just speed were likely to have been necessary for accurate tracking. This finding highlights a need for increased breadth in investigations of motion dazzle camouflage and its mechanisms, specifically into the interactions between types of target movement and the efficacy of dazzle camouflage.

## SUPPLEMENTARY MATERIAL

Supplementary material can be found here

## FUNDING

This work was funded by a Biotechnology and Biological Sciences Research Council, UK (BB/J014400/1), SWBio Doctoral Training Programme studentship to B.H.

## Supplementary Material

Supplementary Data
