# Supplementary material for: Dazzle camouflage, target tracking, and the confusion effect
Source: Behav Ecol. 2016 May 31;27(5):1547–51. doi: 10.1093/beheco/arw081 (PMC5027625; doi:10.1093/beheco/arw081)
Supplement: Supplementary Data [file arw081_supplementary_data.zip › Supplementary_r2.docx]

Hogan et al. Supplementary Material

For those who prefer a null hypothesis testing rather than an information theoretic approach to model assessment, the following results are based on analysis using repeated-measures ANOVA. Target pattern (3 levels), Background (2 levels) and Number (as a quadratic continuous variable) were fixed effects and Participant as a random effect. Each fixed effect was tested against an error term corresponding to the interaction between Participant and that fixed effect, using the function aov in R. The classical ANOVA approach, valid on account of the balanced design of the experiment, also serves as reassurance on the robustness of the results from the GLMM fits in the main text.

A three-way within-subjects ANOVA indicates that the target*number*background interaction was not statistically significant (F(4,60)=2.131, p=.088). The target*background interaction was not statistically significant (F(2,30)=0.851, p=.437). However, the background*number interaction was statistically significant (F(2,30)=3.866, p=.0321), as was the target*number interaction (F(4,60)=4.971, p=.00158). There were significant main effects of target (F(2,30)=10.28, p<.001), background (F(1,15)=7.856, p=.0134), and number (F(2,30)=278.6, p<.001).

Due to the clear trend for increased error in the parallel striped target condition relative to the other colouration conditions, the coding of the factor target was adjusted to assume that orthogonally striped and trinary colourations are identical. With this coding, a three-way within-subjects ANOVA indicates that the three way target*number*background interaction was again not statistically significant (F(2,30)=0.768, p=.473). The target*background interaction was not significantly significant (F(1,15)=1.333, p=.266). However, the background*number interaction was statistically significant (F(2,30)=3.866, p=.0321), as was the target*number interaction (F(2,30)=9.134, p<.001). There was a main effect of target (F(1,15)=19.29, p<.001), background (F(1,15)=7.856, p=.0134), and number (F(2,30)=278.6, p<.001).
